# Comprehensive evaluation of the test for 5′‐/3′‐end mRNA unbalanced expression as a screening tool for ALK and ROS1 fusions in lung cancer

**DOI:** 10.1002/cam4.4686

**Published:** 2022-03-23

**Authors:** Natalia V. Mitiushkina, Alexandr A. Romanko, Elena V. Preobrazhenskaya, Vladislav I. Tiurin, Tatiana I. Ermachenkova, Alexandr S. Martianov, Rimma S. Mulkidjan, Tatiana N. Sokolova, Maksim M. Kholmatov, Ilya V. Bizin, Alexandr O. Ivantsov, Olga S. Yatsuk, Olga A. Zaitseva, Aglaya G. Iyevleva, Ekatherina Sh. Kuligina, Evgeny N. Imyanitov

**Affiliations:** ^1^ Department of Tumor Growth Biology N.N. Petrov Institute of Oncology St.‐Petersburg Russia; ^2^ Department of Medical Genetics St.‐Petersburg Pediatric Medical University St.‐Petersburg Russia; ^3^ Department of Oncology I.I. Mechnikov North‐Western Medical University St.‐Petersburg Russia; ^4^ Department of Oncology I.P. Pavlov St.‐Petersburg State Medical University St.‐Petersburg Russia

**Keywords:** lung cancer, molecular diagnosis, qRT‐PCR, targeted therapy

## Abstract

**Background:**

Despite the progress in the development of next‐generation sequencing (NGS), diagnostic PCR assays remain to be utilized in clinical routine due to their simplicity and low cost. Tests for 5′‐/3′‐end mRNA unbalanced expression can be used for variant‐independent detection of translocations, however, many technical aspects of this methodology require additional investigations.

**Methods:**

Known *ALK*/*ROS1* fusions and 5′‐/3′‐end unbalanced expression were analyzed in 2009 *EGFR* mutation‐negative non‐small cell lung cancer (NSCLC) samples with RT‐PCR tests, which were optimized for the use with FFPE‐derived RNA.

**Results:**

Variant‐specific PCR tests for 4 common *ALK* and 15 common *ROS1* translocations detected 115 (5.7%) and 44 (2.2%) rearrangements, respectively. Virtually all samples with common *ALK* fusions demonstrated some level of 5′/3′ mRNA ends unbalanced expression, and 8 additional NSCLCs with rare *ALK* fusions were further identified by PCR or NGS among 48 cases selected based on *ALK* expression measurements. Interestingly, NSCLCs with unbalanced 5′‐/3′‐end *ALK* expression but without identified *ALK* translocations had elevated frequency of RAS mutations (21/40, 53%) suggesting the role of RAS activation in the alternative splicing of *ALK* gene. In contrast to *ALK*, only a minority of *ROS1* translocation‐positive cases demonstrated unbalanced gene expression, with both 5′‐ and 3′‐end mRNA expression being elevated in most of the samples with translocations. Surprisingly, high *ROS1* expression level was also found to be characteristic for NSCLCs with activating mutations in other tyrosine kinases such as *EGFR*, *ALK*, or *MET*.

**Conclusions:**

Comprehensive ALK analysis can be performed by the test for 5′‐/3′‐end unbalanced expression with minimal risk of missing an ALK rearrangement. In contrast, the use of the test for 5′‐/3′‐end unbalanced expression for the detection of ROS1 fusions is complicated; hence, the utilization of variant‐specific PCR assays for ROS1 testing is preferable.

## Graphical abstract

Known ALK/ROS1 fusions and 5’‐/3’‐end mRNA unbalanced expression were analyzed in 2009 EGFR mutation‐negative non‐small cell lung cancer (NSCLC) samples. Almost all samples with common ALK fusions demonstrated some level of 5’‐/3’‐end mRNA unbalanced expression, while the majority of ROS1 translocation‐positive cases had elevated but balanced gene expression. Interestingly, high ROS1 expression level was also found to be characteristic for NSCLC tumors with activating mutations in other tyrosine kinases, such as EGFR, ALK or MET.

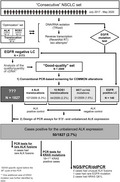

## 1 INTRODUCTION

Lung cancer is a leading cause of cancer‐related deaths worldwide.^1^ Clinical management of non‐small cell lung cancer (NSCLC) underwent significant modifications in the past due to the invention of several groups of targeted drugs. In particular, the development of tyrosine kinase inhibitors (TKIs) led to unprecedented improvement of survival in patients, whose tumors carry an appropriate molecular target. By definition, epidermal growth factor receptor (*EGFR*), ALK receptor tyrosine kinase (*ALK*), ROS proto‐oncogene 1, receptor tyrosine kinase (*ROS1*), B‐Raf proto‐oncogene, serine/threonine kinase (*BRAF*), ret proto‐oncogene (*RET*), MET proto‐oncogene, receptor tyrosine kinase (*MET*), etc. inhibitors are active only in NSCLCs driven by genetic activation of the mentioned kinases. Consequently, the reliable detection of mutations and rearrangements in druggable genes is a key component of the NSCLC treatment.

Some gene‐tailored therapies have been incorporated into the standards of NSCLC treatment, while others still are undergoing final phases of clinical trials and have good chances of getting approval. For the time being, explicit examination of NSCLC tissue samples already require the analysis of activating mutations in *EGFR*, *BRAF*, and KRAS proto‐oncogene, GTPase (*KRAS*) genes, testing for rearrangements in *ALK*, *ROS1*, *RET*, and neurotrophic receptor tyrosine kinases (*NTRK1/2/3*), the detection of *MET* exon 14 skipping mutations, and the assessment of Programmed cell death 1‐ligand 1 (*PD‐L1*) expression. The mutation testing is usually done by allele‐specific PCR and/or gene sequencing. The analysis of rearrangements can be performed by immunohistochemistry (IHC), fluorescence in situ hybridization (FISH), or various molecular genetic assays. *PD‐L1* expression is currently analyzed by IHC. In any scenario, comprehensive molecular analysis of NSCLC requires the involvement of several analytical platforms. This is a limiting factor for NSCLC molecular testing, as many NSCLC patients are diagnosed already at an advanced stage and can provide for the investigation only a small biopsy material.^2^

There is a need for methods that are capable of obtaining all necessary information using a single diagnostic platform. Next‐generation sequencing (NGS) appears to be the best solution, however, it currently has some significant drawbacks such as a high cost, significant turnaround time, and the requirement for sophisticated laboratory infrastructure. There are relatively nonexpensive PCR‐based technologies, which are capable of detecting all NSCLC‐associated alterations. While the testing for recurrent mutations in *EGFR*, *BRAF*, and *KRAS* genes is generally not error‐prone, the analysis of kinase gene fusions presents a challenge due to high variability in the location of breakpoints. Some diagnostic kits (e.g., QFusion *EML4‐AL*K and *KIF5B‐ALK* Fusion Gene Detection Test, DiaCarta, USA; *EGFR/ALK/ROS1* Mutations Detection Kit, AmoyDx, China; *EGFR/ALK/ROS1* Mutations Detection Kit, LCM Genect, Italy) suggest variant‐specific PCR analysis for *ALK* and *ROS1* rearrangements; however, these assays cannot reveal uncommon types of gene fusions.

Alternatively, there are tests for 5′‐/3′‐end unbalanced expression, which are designed for the detection of all actionable rearrangements within a given gene. Oncogenic activation of *ALK*, *ROS1*, *RET*, and *NTRK1/2/3* receptors in NSCLC involves translocation of the kinase portion of the gene under the control of a strong promoter. Consequently, the rearrangement usually results in the elevation of the amount of 3′‐end‐specific transcript, so the PCR‐based evaluation of the 5′‐/3′‐end ratio can be an efficient tool for the identification of tumors with actionable gene fusions.^3^, ^4^, ^5^, ^6^, ^7^, ^8^ Although previous studies demonstrated the feasibility of PCR‐driven detection of translocations in the kinase genes, many technical aspects of this methodology require additional investigations. Several reports described PCR‐based protocols for efficient detection of *ALK* rearrangements.^3^, ^4^, ^5^, ^6^, ^8^ However, these reports did not systematically analyze the actual performance of newly developed assays against large NSCLC collections with rigorously proven *ALK* translocation‐positive and translocation‐negative tumor status. There is a single study describing the utility of the 5′‐/3′‐end unbalanced expression test for the detection of *ROS1* rearrangements.^7^ This investigation revealed only 5 instances of *ROS1* fusions, and the frequency of this event (0.7%) was considerably lower than in other NSCLC series.^9^, ^10^ Real‐world NSCLC diagnostic testing involves formalin‐fixed paraffin‐embedded (FFPE) tumor tissues with varying degrees of degradation of nucleic acids.^11^, ^12^ The development of FFPE‐adjusted protocols, which are capable of processing low‐quality genetic material, is of utmost clinical importance.^12^, ^13^

This study compared several modifications of RT‐PCR tests and suggested protocols, which are the most suitable for the analysis of FFPE tumor tissues. It evaluated the performance of RT‐PCR tests for 5′‐/3′‐end unbalanced expression as a screening tool for *ALK* and *ROS1* fusions in 2009 NSCLC samples. It also analyzed interactions between *ALK* and *ROS1* genes expression and NSCLC‐specific driver events.

## 2 MATERIALS AND METHODS

The study included consecutive NSCLC samples, which were referred for molecular testing to the N.N. Petrov Institute of Oncology (St.‐Petersburg, Russia) from July 2017 to May 2020. Nucleic acids were isolated using the TRIzol Reagent (Thermo Fisher Scientific). The obtained preparations, which contained both DNA and RNA, were further subjected to complementary DNA (cDNA) synthesis using RevertAid Reverse Transcriptase (Thermo Fisher Scientific). The reaction setup included two steps. First, the nucleic acid sample was mixed with deoxyribonucleotide triphosphates (dNTPs) (20 nM each) and primers in a 12.5 μl volume and incubated for 3 min at 70°C, 3 min at 65°C, and 1 min at 60°C in order to denature RNA and anneal gene‐specific primers. The sample was subsequently cooled on ice and the other components of the reaction were added, including 1× Reaction buffer, Thermo Scientific RiboLock RNase inhibitor (20 U), and the reverse transcriptase (200 U). The total volume of the reverse transcription (RT) reaction was 20 μL. After the completion of cDNA synthesis, 40 μl of water was added to the sample. If the first attempt to synthesize cDNA was unsuccessful, we performed the second RT reaction using 1:5 dilution of the initial sample to alleviate the possible influence of reaction inhibitors. The sample was considered unsuitable for analysis only after two failed RNA extractions.

**FIGURE 1:**
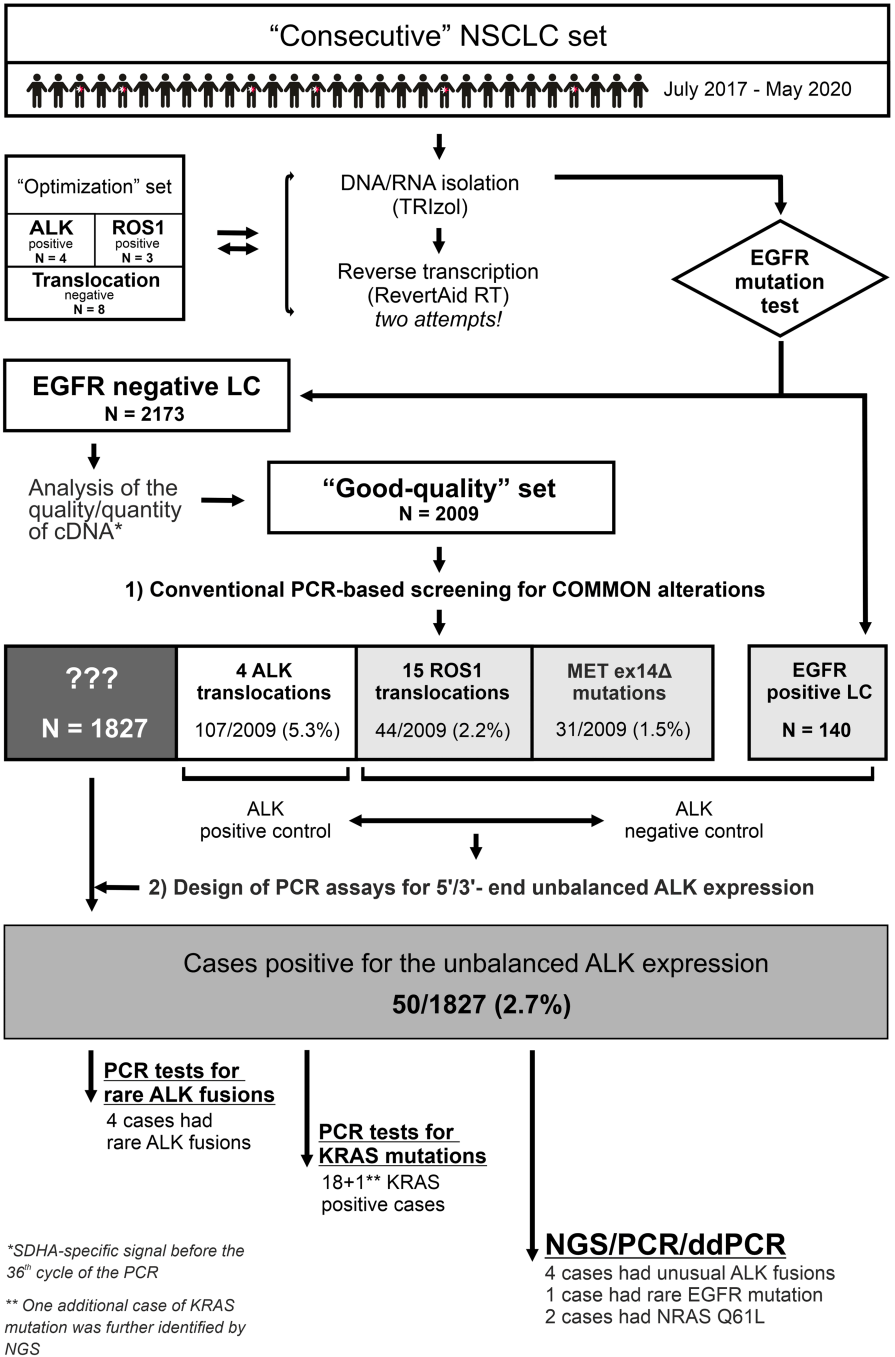
The scheme of the experimental design

The full pipeline of the experiments is shown in Figure 1. Fifteen NSCLC samples (4 *ALK*‐positive, 3 *ROS1*‐positive, and 8 translocation‐negative) were used for comparative analysis of various modifications of the reverse transcription and PCR‐based *ALK*/*ROS1* testing. The aim of these experiments was to find the optimal design of primers for cDNA synthesis and PCR reactions. Some NSCLC specimens referred for the testing were already known to have *EGFR* mutation‐negative status, as they were screened for the presence of *EGFR* mutations in the local laboratories. The remaining NSCLC cases were initially screened for the presence of *EGFR* mutations as described previously.^14^ *EGFR* mutation‐negative specimens (*n* = 2173) were subsequently subjected to the analysis of the quality and quantity of cDNA using succinate dehydrogenase complex flavoprotein subunit A *(SDHA*) as a gene referee. *ALK*/*ROS1* study included NSCLCs (*n* = 2009), which produced SDHA‐specific signals before the 36th cycle of the PCR. *EGFR*‐mutated carcinomas are unlikely to carry *ALK* or *ROS1* rearrangements, therefore, we utilized this category of NSCLC (*n* = 140) as a “negative” control.

The composition of all PCR primers and probes is given in Tables S1. Gene‐specific primer mix for RT reactions consisted of 9 primers corresponding to *ALK*, *ROS1*, and *SDHA* nucleotide sequences (Table S4). These primers were located outside of the respective PCR fragments in order to avoid interference with PCR amplification. Single RT reaction included 10 pM of each gene‐specific primer.

All PCR reactions contained 1× GeneAmp PCR Buffer I (Applied Biosystems, USA), 250 mkM of each dNTP, 200 nM of each primer and probe, 2.5 mM MgCl_2_, and 1 U of TaqM polymerase (AlkorBio, Russia) in a total volume of 20 μl. PCR started from the enzyme activation (95°С, 10 min.) and included 38 cycles (95°С for 15 s followed by 58°С for 1 min.). For calculation purposes, the absence of a detectable PCR curve at the last PCR cycle was considered as the cycle threshold (Ct) equal 38. All PCR reactions were run on the Bio‐Rad CFX96 instrument. MET exon 14 skipping (MET ex14Δ) mutations were tested as described by Mitiushkina et al.^15^ Protocols for *KRAS*, *NRAS*, and *BRAF* mutational analysis were reported previously.^16^, ^17^

Next‐generation sequencing (NGS) was performed using QIAseq‐Targeted RNAscan Panel (Qiagen, Germany) with custom primers, which covered selected exons of the *ALK*, *ROS1*, *RET*, *EGFR*, *KRAS*, *BRAF*, and several other genes (Table S5). The Illumina MiSeq platform was utilized to generate NGS reads.

Digital droplet PCR was carried out on the Bio‐Rad QX100 Droplet Digital PCR system instrument. The following primers and probes were used for the detection of *KRAS G12D* mutation: AAATGACTGAATATAAACTTGT, ATTAGCTGTATCGTCAAGG, FAM‐CCTACGCCACCAG CTC‐BHQ1, and R6G‐CCTACGCCATCAGCTC‐BHQ1.

Sanger sequencing using the GenomeLab GeXP Genetic Analysis System (Beckman Coulter, USA) was performed to confirm the presence of *EZR‐ROS1* (*E10*;*R32*) translocation in one of the samples. PCR product of 111 bp was obtained with the following pair of primers: AGAAGGAGGAGTTGATGCT and TACTCCC TTCTAGTAATTTGG, then the sequencing was performed according to the manufacturer's instruction.

The statistical analysis was performed using R software.^18^ Specifically, the *glm* function from the *stats* package was used to fit binomial logistic regression models, and different functions from the *graphics* and *ggplot2* ^19^ packages were utilized for plotting. All performed tests were two‐sided, and *p* values of <0.5 were considered significant. No adjustments for multiple testing were done in this study, which, in general, has an exploratory character.

## 3 RESULTS

### 3.1 Optimization of cDNA synthesis and PCR conditions

RNA in clinical formalin‐fixed paraffin‐embedded FFPE specimens can be highly degraded; therefore, optimal efficiency of the reverse transcription reaction is critical for the success of the subsequent RNA expression analysis. The conventional reverse transcription protocol^20^ requires the presence of 100 pM random hexanucleotide primers. There are some reports, which suggest that the use of higher concentration of hexanucleotide primers,^21^ or the increase of the length of random primers,^22^ or the use of gene‐specific primers^23^ may result in the improvement of the cDNA production from FFPE‐derived RNA.

Various modifications of the cDNA synthesis were considered and their influence on the efficiency of PCR amplification of *ALK*, *ROS1*, and *SDHA* gene fragments was evaluated. These experiments revealed that the use of 1 nM of hexamers (R6) in RT‐PCR reaction instead of 100 pM resulted in significant improvement of the productivity of PCR reactions (Table 1; Figure S1). The use of decamer random primers (R10) instead of hexamers also provided some advantages. Utilization of gene‐specific primer mix instead of random primers further supported subsequent PCR assays (Table 1; Figure S1).

**TABLE 1:** Changes in PCR cycle threshold (Ct) values as a result of different reverse transcription priming strategies in 15 NSCLC samples

	Difference in PCR Ct values, median (range)		
	R6, 100 pM vs. R6, 1 nM	R6, 100 pM vs. R10, 1 nM	R6, 100 pM vs. gene‐specific primers
*SDHA*	2.8 (1.3–3.7)	3.6 (1.4–5.9)	5 (2–8.3)
*ALK* 3′ test	2.6 (0.3–3.1)	3 (−0.4–4.6)	3.3 (−2.4–6.8)
*ALK* 5′ test	1.3 (0–2.4)	2 (−0.6–5.5)	2.9 (−3.6–5.3)
*ROS1* 3′ test	3 (0.6–4.5)	3.7 (0.4–6)	5.4 (1.2–9.6)
*ROS1* 5′ test	2.7 (1.6–3.4)	3 (0.6–4.9)	3.7 (0.1–6.4)
*ALK/ROS1* variant‐specific PCR	1.1 (0.4–1.3)	1.4 (0.3–2)	2.8 (0.8–4)
Mean Ct change for all tests	2.3	2.8	3.9

The size of PCR fragments is also a limiting factor for the analysis of degraded RNA from FFPE specimens. Our previous studies^5^, ^17^ utilized primers, which generated fragments within the 100–150 bp range. It was evaluated whether further shortening of the length of PCR product increases the efficiency of PCR, and a marginal improvement for some assays was observed (Table S6).

### 3.2 Screening for common *ALK/ROS1* translocations and *MET ex14Δ*

*EGFR* mutation‐negative samples (*n* = 2009) were screened for the four most common *EML4‐ALK* translocations, 15 known *ROS1* translocations, and *MET ex14Δ* mutation. This effort led to the detection of *ALK* fusions in 107/2009 (5.3%), *ROS1* fusions in 44/2009 (2.2%), and *MET ex14Δ* in 31/2009 (1.5%) of NSCLC cases (Table 2). Significant associations were observed between the identified genotypes and such patient characteristics as age, sex, and smoking history (Table 3). Specifically, patients with *ALK* and *ROS1* translocations were younger and patients with *MET ex14Δ* mutation were older than subjects with no identified mutations or the *EGFR*‐positive cases, and all indicated mutations were more frequent in women and never‐smokers. We did not consider associations with tumor histology because there were not enough cases with histological diagnoses other than adenocarcinoma.

**TABLE 2:** Clinical and molecular characteristics of 2009 *EGFR*‐negative NSCLC cases tested for *ALK* and *ROS1* fusions and *MET* ex14 skipping mutation

Characteristic	Number of cases
Age, median (range)	63 (16–85)
Women	629 (31.3%)
Smoking status	
Ever‐smoker	407 (52.5%)
Never‐smoker	368 (47.5%)
Unknown	1234
Histology	
Adenocarcinoma	1731 (99.0%)
Squamous	12 (0.7%)
Other	4 (0.2%)
Not specified	262
*ALK* translocations	107 (5.3%)
*EML4‐ALK (E13;A20)*	49 (45.8%)
*EML4‐ALK (E20;A20)*	14 (13.1%)
*EML4‐ALK (E6;A20)*	41 (38.3%)
*EML4‐ALK (E18;A20)*	3 (2.8%)
*ROS1* translocations	44 (2.2%)
*CD74‐ROS1 (C6;R34)*	23 (52.3%)
*EZR‐ROS1 (E10;R32)*	2 (4.5%)
*EZR‐ROS1 (E10;R34)*	5 (11.4%)
*SDC4‐ROS1 (S2;R32)*	3 (6.8%)
*SLC34A2‐ROS1 (S13;R32)*	7 (15.9%)
*SLC34A2‐ROS1 (S4;R32)*	1 (2.3%)
*TPM3‐ROS1 (T8;R35)*	2 (4.5%)
*TPM3‐ROS1 (T10;R35)* ^a^	1 (2.3%)
*MET* ex14Δ	31 (1.5%)

^a^ This variant was initially detected as *TPM3‐ROS1 (T8;R35)* by real‐time PCR analysis, but further was correctly identified as *TPM3‐ROS1 (T10;R35)* by Sanger sequencing.

**TABLE 3:** Associations between molecular and clinical characteristics

Clinical parameters	Mutational status					*p* value
	*EGFR* mutation (*n* = 140)	*ALK* translocation (*n* = 107)	*ROS1* translocation (*n* = 44)	*MET ex14Δ* (*n* = 31)	No mutations (*n* = 1827)	
Age, median (range)	65 (29–88)	58 (26–80)	59 (30–78)	71 (56–84)	63 (16–85)	1.2·10^−14^ (Kruskal–Wallis rank sum test)
Gender						
Men	31 (22.1%)	39 (36.4%)	11 (25%)	11 (35.5%)	1319 (72.2%)	<2.2·10^−16^ (χ^2^ test)
Women	109 (77.9%)	68 (63.6%)	33 (75%)	20 (64.5%)	508 (27.8%)	
Smoking status						
Ever	9 (42.9%)	10 (24.4%)	2 (20%)	3 (37.5%)	392 (54.7%)	0.00025 (Fisher's Exact Test)
Never	12 (57.1%)	31 (75.6%)	8 (80%)	5 (62.5%)	324 (45.3%)	

The variant‐specific PCR assay utilized in this study included primers for the detection of *EZR‐ROS1* (*E10*;*R32*) translocation. This particular rearrangement was never described in prior investigations, however, it was decided to consider this variant, given that exon 32 of the *ROS1* gene is frequently involved in other translocation events. Two out of 44 *ROS1* fusions, which were identified within this study, were classified as *EZR‐ROS1* (*E10;R32*). The identity of this translocation was confirmed by sequencing in one available sample (Figure S2).

### 3.3 Search for rare ALK variants with the test for 5′‐/3′‐end unbalanced expression

The group of *ALK*‐rearranged NSCLCs (*n* = 107) was used as a “positive control” for subsequent expression experiments, while samples with *EGFR* alterations (*n* = 140), *ROS1* fusions (n = 44), or *MET* exon 14 skipping (*n* = 31) served as a “negative control,” given that the mentioned driver mutations are very unlikely to be coincident with *ALK* fusions.

**FIGURE 2:**
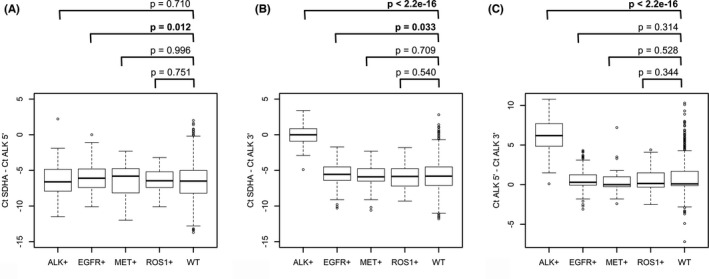
Real‐time PCR expression measurement of 5′‐ and 3′‐ends of *ALK* gene in lung cancer samples with and without common driver mutational events. (A, B) *ALK* 5′‐ and *ALK* 3′‐ends expression relative to the reference gene, *SDHA*. (C) Difference in cycle threshold (Ct) values between PCR reactions for *ALK* 5′‐ and *ALK* 3′‐end fragments. *p*‐ values were calculated using Mann–Whitney *U* test

The expression level of the kinase portion of the *ALK* gene (exons 22 and 23) was compared in *ALK*‐rearranged versus *ALK*‐wild‐type samples (Figure 2). As expected, *EML4*‐*ALK* (*E13;A20*)/(*E20;A20*)/(*E6;A20*)/(*E18;A20*)‐positive samples demonstrated a higher level of expression of 3′‐end *ALK*‐specific RNA compared with other NSCLCs, while no difference was observed in the expression of the 5′‐portion of the gene (exons 9 and 10).

**FIGURE 3:**
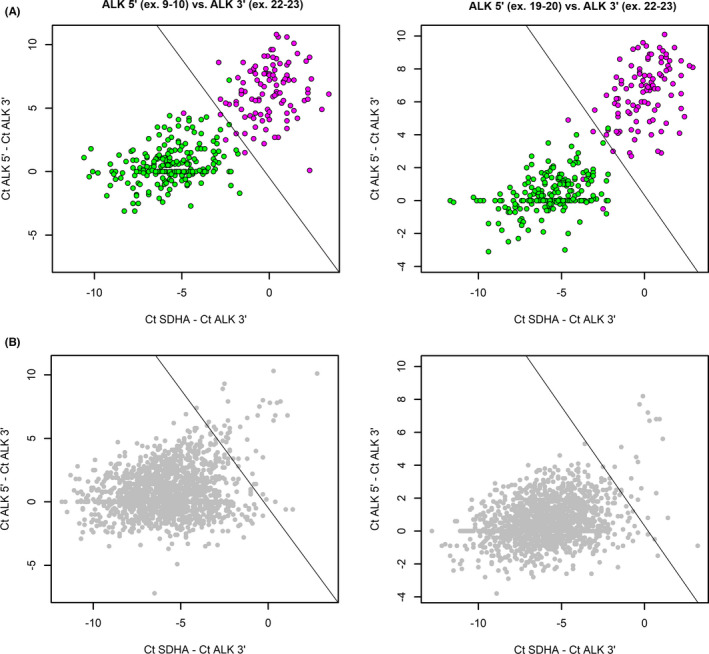
Selection of cases with probable *ALK* translocations based on expression analysis of 5′ and 3′ mRNA ends. (A) Training set was used to fit the logistic regression model. This set included positive samples, where common *ALK* translocation variants were identified (magenta dots), and negative samples, which were shown to possess other common driver mutations in *EGFR*/*ROS1*/*MET* genes (green dots). Lines show thresholds, defined by a logistic regression model with 95% sensitivity. (B) Test set where the same thresholds were applied

Then, two PCR assays for 5′‐/3′‐end unbalanced *ALK* expression were utilized in order to obtain the range of variations within *EML4‐ALK* (*E13;A20*)/(*E20;A20*)/(*E6;A20*)/(*E18;A20*)‐rearranged samples and within presumably *ALK* fusion‐negative (*EGFR‐*/*ROS1‐*/*MET*‐mutated) NSCLCs. One of the assays evaluated the 5′/3′ ratio between exons 9–10 and exons 22–23 portions of the gene; this design is potentially capable to reveal all *ALK* fusions, however, it is likely to be influenced by rearrangement‐independent mechanisms, e.g., by alternative splicing. The fragment corresponding to exons 19–20 was also used as a 5′‐portion. The use of closely located fragments (5′‐end: exons 19 and 20; 3′‐end: exons 22 and 23) is potentially less prone to artifacts; however, this assay will miss the translocations, whose breakpoint is located before the exon 19. We noticed that, occasionally, in cases with relatively balanced 5′/3′‐expression, high ALK expression alone may be indicative of translocation, while in samples with low ALK kinase domain expression, ALK fusions are rarely identified, despite the presence of moderate 5′/3’ mRNA ends imbalance in some of them. Thus, we decided to include both kinase domain (3′‐end) expression relative to the referee gene and the difference between the Ct values for 5′ and 3’ mRNA end fragments as predictors to the binomial logistic regression models. The parameters of the fitted models are given in Table S7. Correct classification of 95% of *ALK*‐positive samples in the training set was taken as a criterion for the selection of thresholds. A sensitivity of 95% was considered to be sufficient for identifying the majority of uncommon *ALK* translocation events in the studied patients. The respective specificities of both models were 99.5%. When these thresholds were applied to *EGFR*/*ROS1*/*MET*‐negative samples, which were lacking common *ALK* translocations, 50 out of 1827 cases (2.7%) were found to be positive by at least one model (Figure 3). Of these 50 cases, 48 contained enough nucleic acids for additional analyses. These 48 cases were analyzed first by additional PCR tests, designed to identify previously reported rare *ALK* fusions (Table S2). With this method, two cases with *EML4‐ALK* (*E2;A20*), one case with *KIF5B‐ALK* (*K17;A20*), and one case with *DCTN1‐ALK* (*D26;A20*) rearrangements were found.

Samples with no identified ALK variants were further considered for NGS analysis. Before performing NGS, all these cases were screened for common mutations in the *KRAS* gene because these genetic events are known to be mutually exclusive with *ALK* translocations. In this cohort, 18 *KRAS*‐positive cases were identified by conventional methods. One additional case of *KRAS* mutation was further identified by NGS. Droplet digital PCR showed that only 2.7% of DNA with the mutation was present in this case, however, an abundant expression of the mutated allele in the tumor tissue made it easily detectable by RNA‐based NGS. Interestingly, two cases of *NRAS Q61L* mutation were also found in this cohort by NGS and were further confirmed by PCR.

NGS detected 4 samples with unusual translocations. In particular, instances of *EML4‐ALK* (E6ins33;A18), *EML4‐ALK* (*E5del10;del44A20*) and rearrangement *HIP1‐ALK* (*H30;A20*) were identified, each in one case. The last sample had four chimeric transcripts: *EML4‐ALK* (*E6ins33;A18*), (*E6ins33;A17*), (*E6;A18*), and (*E6;A17*), likely resulting from alternative splicing. In addition, rare *EGFR* mutation (p.*Val769Leu*) and translocation *CCDC6‐RET* (*C1;R12*) were found by NGS in two other cases. The summary of all results and characteristics for the selected 50 cases are provided in Tables S8 and S9.

### 3.4 *ROS1* rearrangements do not always result in 5′‐/3′‐end unbalanced expression

**FIGURE 4:**
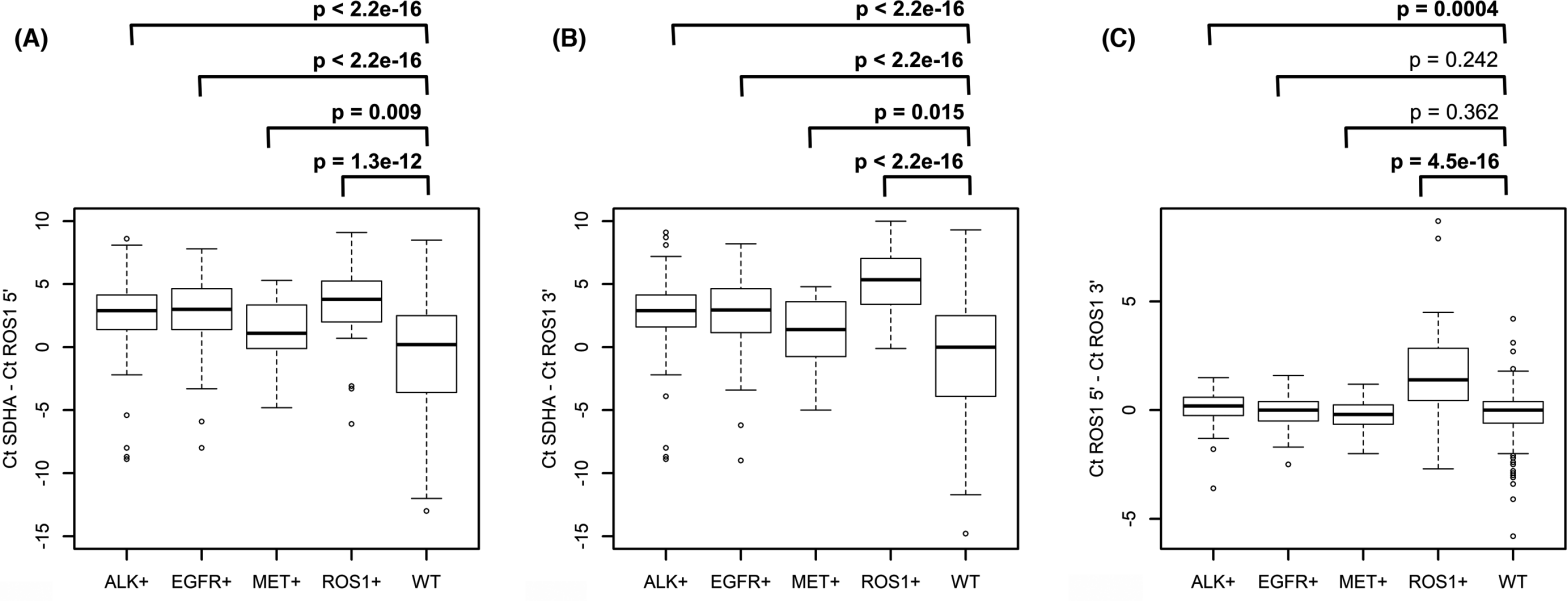
Real‐time PCR expression measurement of 3′‐ and 5′‐ends of *ROS1* gene in lung cancer samples with and without common driver mutational events. (A, B) *ROS1* 3′‐ and *ROS1* 5′‐ends expression relative to the reference gene, *SDHA*. (C) Difference in cycle threshold (Ct) values between PCR reactions for *ROS1* 5′‐ and *ROS1* 3′‐end fragments. p values were calculated using Mann–Whitney *U* test

**FIGURE 5:**
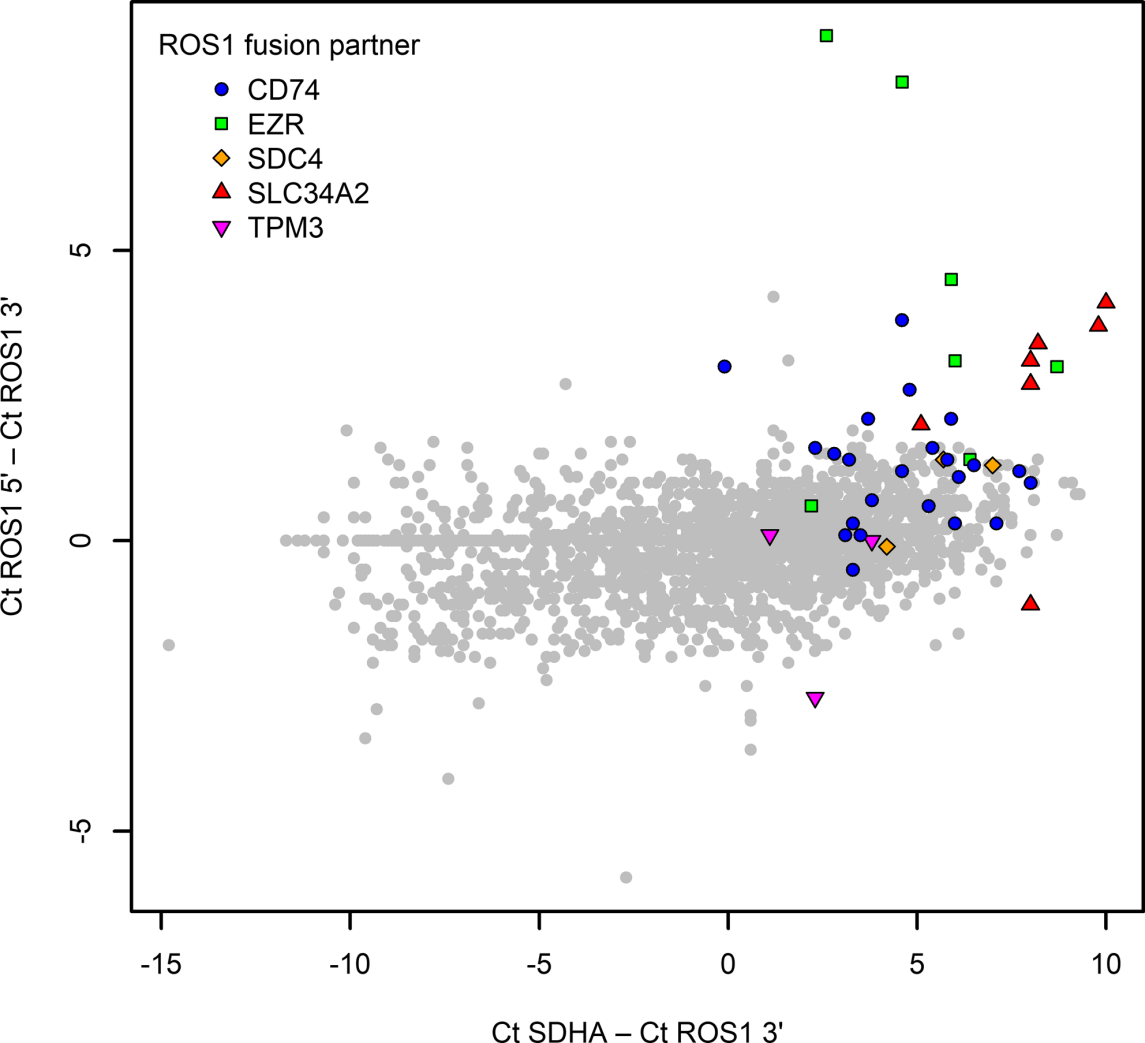
Expression analysis of 5′ and 3’ mRNA ends of *ROS1* gene in *ROS1* translocation‐positive (colored dots) and ‐negative (gray dots) cases

Similar to *ALK*, *ROS1* 5′‐end (exons 17 and 18) and 3′‐end (exons 38 and 39) expressions were analyzed in tumors with established status for *EGFR*, *ALK*, *ROS1*, and *MET* (Figure 4). Surprisingly, *ROS1* expression was increased not only in *ROS1* translocation‐positive cases, but also in tumors with alterations in *EGFR*, *ALK*, and, to a lesser extent, *MET* genes, compared with tumors with no such alterations. Moreover, in *ROS1*‐rearranged tumors, there was a trend to increased expression for both 5′ and 3′ portions of the gene. Similar to *ALK*, *ROS1*‐rearranged tumors demonstrated a trend toward the unbalanced expression of 5′‐ and 3′‐end portions of the gene. However, while the assay for unbalanced expression allowed relatively clear‐cut discrimination between *ALK*‐rearranged and *ALK*‐wild‐type tumors (see above), this difference was less pronounced in the case of *ROS1* translocations (Figures 4C and 5). The test for unbalanced expression appeared to be more effective for the detection of *ROS1* rearrangements involving *EZR* or *SLC34A2* gene partners compared with other types of gene fusions (Kruskal–Wallis test, *p* = 0.002; Figure 5).

It was impossible to develop the logistic regression model for finding the threshold for 5′‐/3′‐end unbalanced expression, which would allow selecting samples for NGS‐based detection of rare translocations. Thus, only tumors with the highest level of ROS1 5′‐/3′‐unbalanced expression (ΔCt > 2) were considered for further study. Two out of three cases satisfying this criterion were available for NGS. *ROS1* translocations were not identified in these two cases. In one of them, however, *BRAF V600E* mutation was found.

## 4 DISCUSSION

Most current molecular diagnostic procedures rely on the use of FFPE tumor tissues. Formalin fixation results in degradation of nucleic acids, thus compromising the performance of genetic tests.^11^, ^12^ This paper describes some technical nuances, which helped to improve the efficacy of PCR‐driven detection of rearranged genes in FFPE‐derived RNA samples. In particular, the advantage of the use of gene‐specific primers compared with random oligonucleotides for the generation of cDNA in the reverse transcription reaction was demonstrated. Random primers may have an increased affinity to GC‐rich versus AT‐rich nucleotide sequences, so their use may potentially lead to an underrepresentation of certain RNA fragments and, consequently, biased results of gene expression quantitation.

This study has several strengths. It evaluated the performance of the PCR test for 5′‐/3′‐unbalanced expression for the detection of *ALK* and *ROS1* rearrangements in a large series of NSCLC samples. In contrast to previous reports, it utilized thoroughly composed extensive collections of translocation‐positive and translocation‐negative controls. The availability of data regarding the status of other NSCLC‐specific alterations has allowed revealing some intriguing biologic relationships.

ALK and ROS1 kinases demonstrate significant similarities with regard to amino acid sequence, mode of genetic alterations, the spectrum of associated tumor types, correlations with clinical characteristics of cancer disease, and patterns of sensitivity to tyrosine kinase inhibitors. In particular, these kinases are usually activated by translocations, and their alterations are relatively common in young or female nonsmokers with NSCLC. However, this study demonstrates substantial differences with regard to RNA expression patterns for these kinases in lung tumors with translocations. This is likely due to distinct baseline mRNA expression levels of the mentioned kinases: while *ALK* is not expressed in normal lung tissue, *ROS1* is present in large amounts in nonaffected bronchial epithelium.^24^

*ALK* rearrangements usually result in clear‐cut RNA overexpression of the kinase portion of the gene. Consequently, the test for 5′‐/3′‐end unbalanced expression is highly informative for the detection of *ALK* fusions, being capable of identifying both common and rare *ALK* fusion variants. It is of interest, 21 (53%) out of 40 tumors, which had evidence for *ALK* 5′‐/3′‐end expression imbalance but were found to be negative for *ALK* translocation, carried mutation in *KRAS* or *NRAS* oncogenes. This frequency clearly exceeds the expected values, as the occurrence of RAS mutations in *EGFR*‐negative lung adenocarcinomas usually falls below 30%.^17^, ^25^, ^26^ Thus, it is tempting to suggest that the presence of *KRAS* or *NRAS* mutations, at least in some lung tumors, is associated with significant overexpression of the kinase‐containing portion of *ALK* gene. Further studies are required to confirm this observation. From the practical point of view, it may be advisable to test *KRAS* and *NRAS* genes for common mutations in all samples with unbalanced 5′/3′‐ends *ALK* expression before proceeding with NGS or FISH analysis. Unfortunately, mutational analysis of KRAS and NRAS genes was not performed for all collected NSCLC samples, and thus we could not evaluate ALK and ROS1 expression in RAS‐positive cases, which is a limitation for this study. Overall, only eight cases with ALK fusions were identified among 48 cases selected on the basis of 5′ and 3’ mRNA end expression analysis (17%). This highlights the need for verification of translocations in samples found positive by the test for 5′/3′‐end unbalanced expression using another method.

In contrast to the excellent performance of the test for 5′/3′‐end unbalanced expression in ALK‐rearranged tumors, the utilization of this approach for the analysis of *ROS1* rearrangements is complicated if at all feasible. This study revealed that only some of *ROS1* translocation variants produced disequilibrium between 5′ and 3’ mRNA ends expression. This observation is contradictory to the results obtained by Kalla et al. (2016), who reported 99% accuracy of their 5′/3′‐end *ROS1* mRNA unbalanced expression assay.^7^ However, the study of Kalla et al. (2016) identified only 5 *ROS1*‐rearranged cases, and therefore could not consider the diversity of various *ROS1* translocation variants. In another study, which utilized the Nanostring technology, only three out of seven samples with ROS1 fusions could be detected solely on the basis of analysis of ROS1 mRNA expression patterns, which is more consistent with the results presented here.^27^ Our study also identified novel recurrent translocation *EZR‐ROS1 (E10;R32)*, which deserves to be incorporated in variant‐specific PCR *ROS1* assays.

Interestingly, although *ROS1*‐rearranged tumors demonstrated an elevated level of *ROS1*‐specific mRNA in this study, the increase in the expression of both 5′‐ and 3′‐portions of the *ROS1* gene was observed, perhaps suggesting some interaction with the remaining wild‐type copy of the gene. Even more intriguing, this study demonstrated that *ROS1* mRNA overexpression is characteristic for all tumors carrying activated tyrosine kinases, for example, *ALK, ROS1, EGFR*, or *MET* (Figure 4). This observation is in good agreement with the study of Zhao et al. (2018), who showed that ROS1 IHC‐positive tumors, which are negative for *ROS1* translocations by FISH, often possess *EGFR* mutations or *ERBB2* aberrations.^28^ One could speculate that in tumors, whose growth is dependent on the constant activation of one of the receptor tyrosine kinase, concomitant overexpression of other tyrosine kinase receptors may provide an additional survival advantage. In line with this, elevated expression of *MET* gene in *ALK*/*ROS1*/*EGFR*‐positive tumors was also observed (Figure S3).

Only selected tumors from this NSCLC series were subjected to NGS analysis. NGS analysis, being a gold standard for the detection of gene rearrangements, is expensive and requires a higher quality of nucleic acids compared with PCR. This investigation did not involve comprehensive NGS testing for all NSCLC samples as well as did not utilize IHC or FISH analysis of ALK/ROS1 fusions. This is a limitation of the study because there is a probability that some ALK or ROS1 rearrangements were missed. Nevertheless, given the frequency of identified gene fusions and good concordance between various approaches utilized in this report, one can assume that the rate of false‐negative results in this study is low.

The advantages of PCR‐based diagnostic methods include their general compatibility with hospital‐based laboratory settings and a relatively low cost. The size of biopsied FFPE tissues is usually vanishingly small. It is essential to note that despite this study involved a high number of individual PCR reactions per each DNA/RNA preparation, one or two FFPE sections were sufficient to obtain the necessary amount of nucleic acids. Where needed, EGFR and/or KRAS mutation testing was also performed on the same sample. Furthermore, PCR reactions for individual translocation variants can be effectively multiplexed (data not shown), thus requiring even less biological material. In contrast, the utilization of IHC or FISH requires serial tissue sectioning, therefore, the size of the available tumor sample is critical for the success of these methods. The limitation of the proposed methodology is the need of performing NGS or FISH analysis for those NSCLCs, in which unbalanced ALK expression was not accompanied by the identification of one of the known fusion variants. Also, PCR‐based technology is arguably not efficient in identifying rare fusion variants for ROS1 oncogene. While there is hope that multigene NGS testing will become a universal upfront diagnostic procedure in the near future, the suggested PCR protocol is a viable contemporary alternative to NGS, given the current cost‐ and labor‐related limitations of massive parallel sequencing.

There are several novelties of this study. For the first time, this investigation systematically utilized a quantitative approach to evaluate the performance of the test for unbalanced 5′‐/3′‐end expression; these experiments determined relevant thresholds and revealed limitations of this method in detecting gene fusions. It is generally believed that all actionable rearrangements in NSCLC can be revealed using the more or less universal methodology, however, this report emphasizes differences with regard to ALK and ROS1 testing. Similar research needs to be undertaken for other druggable translocations, for example, alterations affecting RET and NTRK1/2/3 genes.

There are several conclusions from this study. The use of gene‐specific primers in the reverse transcription reactions allows for improving the performance of PCR‐based detection of translocations in FFPE samples. If the use of gene‐specific oligonucleotides is not feasible, it is advisable to utilize high concentrations of random decamer primers for cDNA synthesis. Comprehensive *ALK* analysis can be performed by the test for 5′‐/3′‐end unbalanced expression with minimal risk of missing an *ALK* rearrangement. However, positive *ALK* status should be confirmed by additional tests (variant‐specific PCR, NGS, or FISH), as some NSCLCs, particularly those with mutations in the *RAS* genes, may demonstrate substantial differences between 5′ and 3’ mRNA ends expression in the absence of translocation. In contrast, the use of the test for 5′‐/3′‐end unbalanced expression for the detection of ROS1 fusions is complicated; hence, the utilization of variant‐specific PCR assays for *ROS1* testing is preferable. High *ROS1* mRNA expression is characteristic not only for *ROS1*‐rearranged NSCLCs, but also for tumors with driver mutations in other tyrosine kinase receptor genes (*EGFR*, *ALK*, and *MET*), therefore, it is not a reliable indicator of *ROS1* translocations.

## CONFLICT OF INTEREST

The authors declare no conflict of interest.

## AUTHOR CONTRIBUTION

Mitiushkina N.V., Imyanitov E.N.—conception and design of the study; Romanko A.A., Preobrazhenskaya E.V., Tiurin V.I., Ermachenkova T.I., Martianov A.S., Mulkidjan R.S., Sokolova T.N., Ivantsov A.O., Yatsuk O.S., and Zaitseva O.A.—acquisition of the experimental data; Mitiushkina N.V., Romanko A.A., Kholmatov M.M., Bizin I.V., Iyevleva A.G., and Kuligina E.Sh.—analysis and interpretation of data; Mitiushkina N.V., Imyanitov E.N., and Kuligina E.Sh.—drafting the manuscript. All authors have revised the manuscript and gave the final approval of the version to be published.

## ETHICS STATEMENT

The investigation was conducted in accordance with the Helsinki Declaration and approved by the local ethics committee. Patients provided written informed consent.

## Supporting information


Table S1
Table S2
Table S3
Table S4
Table S5
Table S6
Table S7
Table S8
Table S9
Figure S1
Figure S2
Figure S3

## Data Availability

The data that support the findings of this study are available from the corresponding author upon reasonable request
