# Supplementary material for: Comprehensive evaluation of the test for 5′‐/3′‐end mRNA unbalanced expression as a screening tool for ALK and ROS1 fusions in lung cancer
Source: Cancer Med. 2022 Mar 23;11(17):3226–37. doi: 10.1002/cam4.4686 (PMC9468436; doi:10.1002/cam4.4686)
Supplement: Supplementary file 1 — Table S1 Table S2 Table S3 Table S4 Table S5 Table S6 Table S7 Table S8 Table S9 Figure S1 Figure S2 Figure S3 [file CAM4-11-3226-s001.docx]

**Supplemental Tables**

Table S1. PCR primers and probes used for *ALK* and *ROS1* expression tests

|  | Oligonucleotide name | Sequence | PCR fragment length, bp |
| --- | --- | --- | --- |
| Test for RNA integrity and amount (amplification of a housekeeping gene fragment) | | | |
| SDHA  (exons 5-6) | SDHAex5F | CCACTCGCTATTGCACACC | 78 |
|  | SDHAex6Rlna | TCCAAGGCAA[LNA-A]ATACTCCAC |  |
|  | SDHAex5-6P | R6G-AAGGTCTCTGCGATATGATACC-BHQ2 |  |
| Test 1 for ALK 5’/3’-unbalanced expression (multiplex reaction) | | | |
| ALK 3’-fragment (exons 22-23) | ALKex22F | GATTTCCTCATGGAAGCCCT | 66 |
|  | ALKex23R | CCCAATGCAGCGAACAATGT |  |
|  | ALKex22-23P | R6G-ATCATCAGCAAATTCAACCACCAG-BHQ2 |  |
| ALK 5’-fragment  (exons 9-10) | ALKex9F | GATGGTGTTGCCTCTCCTC | 63 |
|  | ALKex10R | CCCCACCATGCGACCATC |  |
|  | ALKex9-10P | FAM-ATGTGTCTGACAGGTTCTGGCTG-BHQ1 |  |
| Test 2 for ALK 5’/3’-unbalanced expression (multiplex reaction) | | | |
| ALK 3’-fragment (exons 22-23) | ALKex22F | GATTTCCTCATGGAAGCCCT | 66 |
|  | ALKex23R | CCCAATGCAGCGAACAATGT |  |
|  | ALKex22-23P | R6G-ATCATCAGCAAATTCAACCACCAG-BHQ2 |  |
| ALK 5’-fragment  (exons 19-20) | ALKex19F | TCTCCGGCATCATGATTGTG | 73 |
|  | ALKex20R | GGGCTCTGCAGCTCCATCT |  |
|  | ALKex20P | FAM-ATGGCTTGCAGCTCCTGGTGCT-BHQ1 |  |
| Test for ROS1 5’/3’-unbalanced expression (multiplex reaction) | | | |
| ROS1 3’-fragment (exons 22-23) | ROS1ex38F | GAGACCTTCTTACTTATTTGCG | 71 |
|  | ROS1ex39R | GTCAACCAAGGTGAGTAAAGG |  |
|  | ROS1ex38-39P | R6G-AGCCCGGATGGCAACGTTTTATG-BHQ2 |  |
| ROS1 5’-fragment  (exons 17-18) | ROS1ex17F | TGTACACAGCTGTTCTTCGG | 66 |
|  | ROS1ex18R | TCCAGGCTGCAAATTCTGTG |  |
|  | ROSex17-18P | FAM-ACAGAGCACTGGGGATACCACC-BHQ1 |  |

Table S2. PCR primers and probes sequences for *ALK* translocation variants detection

| Fusion | Breakpoint pattern | Breakpoint’s details (cDNA) | Variant name | Oligonucleotide name | Sequence | PCR fragment length, bp |
| --- | --- | --- | --- | --- | --- | --- |
| *EML4-ALK* | *(E13;A20)^1-2^* | -//- | 1 | EML4ex13F | TGGAGCAAAACTACTGTAGAG | 101 |
|  |  |  |  | ALKex20R | GGGCTCTGCAGCTCCATCT |  |
|  |  |  |  | ALKex20P | FAM-ATGGCTTGCAGCTCCTGGTGCT-BHQ1 |  |
|  |  | *(E13;ins69A20)* | 6 | EML4ex13F | TGGAGCAAAACTACTGTAGAG | 76 |
|  |  |  |  | ALKint19v6R | TGGCCCTTGAAGCACTACAC |  |
|  |  |  |  | ALKex20v6P | GGAAAGGACCTAAAGGAAGTGGC |  |
|  | *(E20;A20)^1,3^* | -//-  *(E20;ins18A20)* | 2  2a | EML4ex20F | CTAACTCGGGAGACTATGAAAT | 85  103 |
|  |  |  |  | ALKex20R | GGGCTCTGCAGCTCCATCT |  |
|  |  |  |  | ALKex20P | FAM-ATGGCTTGCAGCTCCTGGTGCT-BHQ1 |  |
|  | *(E6;A20)^4-6^* | -//-  *(E6ins18;A20)*  *(E6ins33;A20)*  *(E6ins11;A20)* | 3  3a  3b  - | EML4ex6F | CATAAAGATGTCATCATCAACCA | 80  98  113  91 |
|  |  |  |  | ALKex20R | GGGCTCTGCAGCTCCATCT |  |
|  |  |  |  | ALKex20P | FAM-ATGGCTTGCAGCTCCTGGTGCT-BHQ1 |  |
|  | *(E14;A20)^2,7,8^* | *(E14;ins11del49A20)*  *(E14;del12A20)*  *(E14;del36A20)* | 4  7  7a | EML4ex14F | CTGTGGGATCATGATCTGAAT | 89  115  91 |
|  |  |  |  | ALKex20R | GTCGAGGTGCGGAGCTTG |  |
|  |  |  |  | ALKex20P | FAM-CTTGCTCAGCTTGTACTCAGGGC-BHQ1 |  |
|  | *(E2;A20)^7^* | -//- | 5a | EML4ex2F | CTGAAGATCATGTGGCCTCA | 100 |
|  |  |  |  | ALKex20R | GGGCTCTGCAGCTCCATCT |  |
|  |  |  |  | ALKex20P | FAM-ATGGCTTGCAGCTCCTGGTGCT-BHQ1 |  |
|  |  | *(E2;ins117A20)* | 5b | EML4ex2F | CTGAAGATCATGTGGCCTCA | 108 |
|  |  |  |  | ALKint19v5bR | TACACAGGCCACTTCCTACA |  |
|  |  |  |  | ALKv5bP | FAM-CAGTCTCAAGTAAAGGTTCAGAGC-BHQ1 |  |
|  | *(E15;A20)^9^* | *(E15del60; del71A20)* | «4» | EML4ex15F | GTAGCAGAAGGAAAGGCAG | 80 |
|  |  |  |  | ALKex20RforE15;A20 | TAGTTGGGGTTGTAGTCGGT |  |
|  |  |  |  | ALKex20PforE15;A20 | FAM-ATTTTTAGTAGGCAAGCTCCGCAC-BHQ1 |  |
|  | *(E18;A20)^10^* | -//- | «5» | EML4ex18F | ACACAGACGGGAATGAACAG | 100 |
|  |  |  |  | ALKex20R | GGGCTCTGCAGCTCCATCT |  |
|  |  |  |  | ALKex20P | FAM-ATGGCTTGCAGCTCCTGGTGCT-BHQ1 |  |
| *KIF5B-ALK* | *(K15;A20)^11^* | -//- | - | KIF5Bex15F | GCTGTGGGAAATAATGATGTAA | 79 |
|  |  |  |  | ALKex20R | GGGCTCTGCAGCTCCATCT |  |
|  |  |  |  | ALKex20P | FAM-ATGGCTTGCAGCTCCTGGTGCT-BHQ1 |  |
|  | *(K17;A20)^12^* | -//- | - | KIF5Bex17F | CGATGCCCTCAGTGAAGAAC | 96 |
|  |  |  |  | ALKex20R | GGGCTCTGCAGCTCCATCT |  |
|  |  |  |  | ALKex20P | FAM-ATGGCTTGCAGCTCCTGGTGCT-BHQ1 |  |
|  | *(K24;A20)^2^* | -//- | - | KIF5Bex24F | CGCATAAAGGAAGCAGTCAG | 116 |
|  |  |  |  | ALKex20R | GGGCTCTGCAGCTCCATCT |  |
|  |  |  |  | ALKex20P | FAM-ATGGCTTGCAGCTCCTGGTGCT-BHQ1 |  |
| *KLC1-ALK* | *(K9;A20)^13^* | -//- | - | KLC1ex9F | TCTCACTCGTGCACATGAAAG | 96 |
|  |  |  |  | ALKex20R | GGGCTCTGCAGCTCCATCT |  |
|  |  |  |  | ALKex20P | FAM-ATGGCTTGCAGCTCCTGGTGCT-BHQ1 |  |
| *SQSTM1-ALK* | *(S5;A20)^14^* | -//- | - | SQSTM1ex5F | TGAAGAACGTTGGGGAGAGT | 100 |
|  |  |  |  | ALKex20R | GGGCTCTGCAGCTCCATCT |  |
|  |  |  |  | ALKex20P | FAM-ATGGCTTGCAGCTCCTGGTGCT-BHQ1 |  |
| *DCTN1-ALK* | *(D26;A20)^14^* | -//- | - | DCTN1ex26F | CTGGTCTCTGGCATTGCTG | 77 |
|  |  |  |  | ALKex20R | GGGCTCTGCAGCTCCATCT |  |
|  |  |  |  | ALKex20P | FAM-ATGGCTTGCAGCTCCTGGTGCT-BHQ1 |  |
| *HIP1-ALK* | *(H21;A20)^15^* | -//- | - | HIP1ex21F | TGCCTCAGAGCCCCACCT | 86 |
|  |  |  |  | ALKex20R | GGGCTCTGCAGCTCCATCT |  |
|  |  |  |  | ALKex20P | FAM-ATGGCTTGCAGCTCCTGGTGCT-BHQ1 |  |
| *HIP1-ALK* | *(H28;A20)^16^* | -//- | - | HIP1ex28F | TCCGGCAAATCACAGATCGAA | 83 |
|  |  |  |  | ALKex20R | GGGCTCTGCAGCTCCATCT |  |
|  |  |  |  | ALKex20P | FAM-ATGGCTTGCAGCTCCTGGTGCT-BHQ1 |  |
| *TPR-ALK* | *(T15;A20)^17^* | -//- | - | TPRex15F | GAGTTGCCATTCCATTACAT | 76 |
|  |  |  |  | ALKex20R | GGGCTCTGCAGCTCCATCT |  |
|  |  |  |  | ALKex20P | FAM-ATGGCTTGCAGCTCCTGGTGCT-BHQ1 |  |
| *SEC31A-ALK* | *(S21;A20)^18^* | -//- | - | SEC31Aex21F | TCATATGCACACCCAGGTAC | 96 |
|  |  |  |  | ALKex20R | GGGCTCTGCAGCTCCATCT |  |
|  |  |  |  | ALKex20P | FAM-ATGGCTTGCAGCTCCTGGTGCT-BHQ1 |  |
| *SOCS5-ALK* | *(S2;A20)^19^* | -//- |  | SOCS5ex2F | AACGAGAACCAGTCAAGGC | 81 |
|  |  |  |  | ALKex20R | GGGCTCTGCAGCTCCATCT |  |
|  |  |  |  | ALKex20P | FAM-ATGGCTTGCAGCTCCTGGTGCT-BHQ1 |  |

^1^Soda M. et al. Nature 2007, 448:561-566. ^2^Takeuchi K. et al. Clin Cancer Res 2009, 15:3143-3149. ^3^Takahashi T. et al. Ann Surg Oncol 2010, 17:889-897. ^4^Choi Y.L. et al. Cancer Res 2008, 68:4971-4976. ^5^Wang R. et al. Clin Cancer Res 2012, 18:4725-4732. ^6^Rikova K. et al. Cell 2007, 131:1190-1203. ^7^Takeuchi K. et al. Clin Cancer Res 2008, 14:6618-6624. ^8^Yoshida A. et al. Lung Cancer 2011, 72:309-315. ^9^Koivunen J.P. et al. Clin Cancer Res 2008, 14:4275-4283. ^10^Wong D.W. et al. Cancer 2009, 115:1723-1733. ^11^Wong D.W. et al. Cancer 2011, 117:2709-2718. ^12^Takeuchi K. et al. Nat Med 2012, 18:378-81. ^13^Togashi Y. et al. PloS One 2012, 7:e31323. ^14^Iyevleva A.G. et al. Cancer Lett 2015, 362:116-121. ^15^Hong M. et al. J Thorac Oncol 2014, 9:419-422. ^16^Fang D.D. et al. J Thorac Oncol 2014, 9:285-294. ^17^Choi Y.L. et al. J Thorac Oncol 2014, 9:563-566. ^18^Kim R.N. et al. Cancer Res Treat 2016, 48:398-402. ^19^Drilon A. et al. Clin Cancer Res 2015, 21:3631-3639.

Table S3. PCR primers and probes sequences for ROS1 translocation variants detection

| Fusion | Breakpoint pattern | Breakpoint’s details (cDNA) | Oligonucleotide name | Sequence | PCR fragment length, bp |
| --- | --- | --- | --- | --- | --- |
| *CD74-ROS1* | *(C6;R32)^1^* | -//- | CD74ex6F | CCACTGACGCTCCACCGAA | 69 |
|  |  |  | ROS1ex32R | TACTCCCTTCTAGTAATTTGG |  |
|  |  |  | ROS1ex32P | FAM-ATGCCTGGTTTATTTGGGACTC-BHQ1 |  |
|  | *(C6;R34)^1,2^* | -//- | CD74ex6F | CCACTGACGCTCCACCGAA | 85 |
|  |  |  | ROS1ex34Rlna | ACAACCAGAA[LNA-A]TATTCCAACTA |  |
|  |  |  | ROS1ex34Plna | FAM-CTTGTTTCTGGTATCCAA[LNA-A]AATCA-BHQ1 |  |
| *SLC34A2-ROS1* | *(S4;R32)^2,3^* | -//- | SLC34A2ex4F | GGATTGGGAGATTGATTTTACT | 138 |
|  |  |  | ROS1ex32R | TACTCCCTTCTAGTAATTTGG |  |
|  |  |  | ROS1ex32P | FAM-ATGCCTGGTTTATTTGGGACTC-BHQ1 |  |
|  | *(S4;R34)^2^* | -//- | SLC34A2ex4F | GGATTGGGAGATTGATTTTACT | 154 |
|  |  |  | ROS1ex34Rlna | ACAACCAGAA[LNA-A]TATTCCAACTA |  |
|  |  |  | ROS1ex34Plna | FAM-CTTGTTTCTGGTATCCAA[LNA-A]AATCA-BHQ1 |  |
|  | *(S13;R32)^1^* | (S13del47;R32) | SLC34A2ex13F | CATTAGCAGAGAGGCTCAG | 68 |
|  |  |  | ROS1ex32R | TACTCCCTTCTAGTAATTTGG |  |
|  |  |  | ROS1ex32P | FAM-ATGCCTGGTTTATTTGGGACTC-BHQ1 |  |
|  | *(S13;R34)^1^* | *(S13del47;R34)* | SLC34A2ex13F | CATTAGCAGAGAGGCTCAG | 84 |
|  |  |  | ROS1ex34Rlna | ACAACCAGAA[LNA-A]TATTCCAACTA |  |
|  |  |  | ROS1ex34Plna | FAM-CTTGTTTCTGGTATCCAA[LNA-A]AATCA-BHQ1 |  |
| *SDC4-ROS1* | *(S2;R32)^1^* | -//- | SDC4ex2F | ACCAGACGATGAGGATGTAG | 99 |
|  |  |  | ROS1ex32R | TACTCCCTTCTAGTAATTTGG |  |
|  |  |  | ROS1ex32P | FAM-ATGCCTGGTTTATTTGGGACTC-BHQ1 |  |
|  | *(S2;R34)^4^* | -//- | SDC4ex2F | ACCAGACGATGAGGATGTAG | 138 |
|  |  |  | ROS1ex34Rlna | ACAACCAGAA[LNA-A]TATTCCAACTA |  |
|  |  |  | ROS1ex34Plna | FAM-CTTGTTTCTGGTATCCAA[LNA-A]AATCA-BHQ1 |  |
|  | *(S4;R32)^1^* | -//- | SDC4ex4F | CCGTTGAAGAGAGTGAGGAT | 141 |
|  |  |  | ROS1ex32R | TACTCCCTTCTAGTAATTTGG |  |
|  |  |  | ROS1ex32P | FAM-ATGCCTGGTTTATTTGGGACTC-BHQ1 |  |
|  | *(S4;R34)^1^* | -//- | SDC4ex4F | CCGTTGAAGAGAGTGAGGAT | 157 |
|  |  |  | ROS1ex34Rlna | ACAACCAGAA[LNA-A]TATTCCAACTA |  |
|  |  |  | ROS1ex34Plna | FAM-CTTGTTTCTGGTATCCAA[LNA-A]AATCA-BHQ1 |  |
| *TPM3-ROS1* | *(T8;R35)^1^* | -//- | TPM3ex8F | GAAAAGACAATTGATGACCTG | 75 |
|  |  |  | ROS1ex35R | TGTCACCCCTTCCTTGGCA |  |
|  |  |  | ROS1ex35Plna | FAM-TCTGGCATAGAAGATT[LNA-A]AAGAATC-BHQ1 |  |
| *EZR-ROS1* | *(E10;R32)* | -//- | EZRex10F | AGACAAAGAAGGCAGAGAGA | 99 |
|  |  |  | ROS1ex32R | TACTCCCTTCTAGTAATTTGG |  |
|  |  |  | ROS1ex32P | FAM-ATGCCTGGTTTATTTGGGACTC-BHQ1 |  |
|  | *(E10;R34)^1^* | -//- | EZRex10F | AGACAAAGAAGGCAGAGAGA | 85 |
|  |  |  | ROS1ex34Rlna | ACAACCAGAA[LNA-A]TATTCCAACTA |  |
|  |  |  | ROS1ex34Plna | FAM-CTTGTTTCTGGTATCCAA[LNA-A]AATCA-BHQ1 |  |
| *FIG-ROS1* | *(F7;R35)^5^* | -//- | FIGex7F | GTTTGTACCTTGATGAGTTAGA | 134 |
|  |  |  | ROS1ex35R | TGTCACCCCTTCCTTGGCA |  |
|  |  |  | ROS1ex35Plna | FAM-TCTGGCATAGAAGATT[LNA-A]AAGAATC-BHQ1 |  |
| *LRIG3-ROS1* | *(L16;R35)^1^* | -//- | LRIG3ex16F | GGATGGGTACGTGTCTTCAG | 142 |
|  |  |  | ROS1ex35R | TGTCACCCCTTCCTTGGCA |  |
|  |  |  | ROS1ex35Plna | FAM-TCTGGCATAGAAGATT[LNA-A]AAGAATC-BHQ1 |  |

^1^Takeuchi K. et al. Nat Med 2012, 18: 378-81. ^2^Rikova K. et al. Cell 2007, 131: 1190-1203. ^3^Bergethon K. et al. J Clin Oncol 2012, 30: 863-870. ^4^Davies K.D. et al. Clin Cancer Res 2012, 18: 4570-4579. ^5^Suehara Y. et al. Clin Cancer Res 2012, 18: 6599-6608.

Table S4. Gene-specific primers used in RT-PCR

| Oligonucleotide name | Sequence |
| --- | --- |
| SDHAex6_RT | CACTCCCCGTTCTCCATCA |
| ALKex23_RT | AGCTCCAGCAGGATGAACC |
| ALKex10_RT | CACGATGGCTCTGGATCCTT |
| ALKex20_RT | TTGCTCAGCTTGTACTCAGG |
| ROS1ex39_RT | ATATCTACACACAGGTCTACA |
| ROS1ex18_RT | CAGTGCATTCTGGGAAATTTC |
| ROS1ex32_RT | GCTTTCTCCCACTGTATTGA |
| ROS1ex34_RT | AGTGGGATTGTAACAACCAGA |
| ROS1ex35_RT | CTTTGTCTTCGTTTATAAGCAC |

Table S5. NGS panel detail.

| Gene | chr | Transcript | Exons | Fusion / junction side | Target sites | Number of primers per target region |
| --- | --- | --- | --- | --- | --- | --- |
| *ALK* | 2 | ENST00000389048.8 | 17, 18, 19, 20, 21, 22, 23 | 3' |  | 2 |
| *ALK* | 2 | ENST00000389048.8 | 20, intronic | 3' | 29223456, 29223370, 29224729, 29223596, 29223510, 29223644 | 2 |
| *ROS1* | 6 | ENST00000368508.7 | 32, 35, 36 | 3' |  | 2 |
| *RET* | 10 | ENST00000355710.8 | 7, 8, 11, 12 | 3' |  | 2 |
| *NTRK1* | 1 | ENST00000368196.7 | 9, 10, 12 | 3' |  | 2 |
| *NTRK2* | 9 | ENST00000376214.5 | 13, 15, 16 | 3' |  | 2 |
| *NTRK3* | 15 | ENST00000394480.6 | 14, 15 | 3' |  | 2 |
| *NRG1* | 8 | ENST00000287842.7 | 2, 4, 6 | 3' |  | 2 |
| *PDGFRb* | 5 | ENST00000261799.9 | 10, 12 | 3' |  | 2 |
| *MET* | 7 | ENST00000318493.11 | 15 | 3' |  | 1 |
| *EWSR1* | 22 | ENST00000397938.6 | 7, 8, 10 | 5' |  | 1 |
| *KRAS* | 12 | ENST00000256078.10 | 2, 3, 4 | point mutation target site | 25245350, 25227343, 25225628 | 1 |
| *NRAS* | 1 | ENST00000369535.5 | 2, 3, 4 | point mutation target site | 114716126, 114713909, 114709586 | 1 |
| *BRAF* | 7 | ENST00000646891.1 | 15 | point mutation target site | 140753337 | 1 |
| *EGFR* | 7 | ENST00000275493.7 | 18, 19, 20, 21 | point mutation target site | 55174015, 55174795, 55181309_55181378, 55191831 | 1 |

Table S6. Difference in Ct values between PCR tests with longer and shorted amplified fragments

| Fragment | Old fragment length, bp | New fragment length, bp | Median difference between old and new Ct (min-max) in 15 samples |
| --- | --- | --- | --- |
| ALK 5’-fragment (exons 9-11) | 135 | 63 | 0.7 (-0.3-2) |
| ALK 3’-fragment (exons 22-23) | 132 | 66 | 3.4 (2.8-5) |
| EML4-ALK (E13;A20) | 134 | 101 | 1.2 (0.8-1.4) |
| EML4-ALK (E20;A20) | 118 | 85 |  |
| EML4-ALK (E18;A20) | 133 | 100 |  |
| SDHA | 102 | 79 | 0.6 (0.3-1.4) |

Ct – PCR cycle threshold; bp – base pairs.

Table S7. Multivariate logistic regression parameters

| Predictor | Coefficient | p-value |
| --- | --- | --- |
| Model 1 (AUC 99.81%) | | |
| Ct SDHA - Ct ALK 3' | 1.9198 | **7.06·10^-05^** |
| Ct ALK 5'(ex9-10) - Ct ALK 3' | 1.0318 | **0.000764** |
| Intercept | 1.2159 | 0.424 |
| Model 2 (AUC 99.73%) | | |
| Ct SDHA - Ct ALK 3' | 1.5291 | **0.000606** |
| Ct ALK 5'(ex19-20) - Ct ALK 3' | 1.0549 | **0.000485** |
| Intercept | 0.8704 | 0.614336 |

Ct – PCR cycle threshold

Table S8. PCR and NGS results for the 50 NSCLC cases with abnormal patterns of *ALK* expression

|  | Age | Sex | Histology | Smoking status | Ct ALK 5' (ex9-10) - Ct ALK 3' | Ct ALK 5' (ex19-20) - Ct ALK 3' | Ct SDHA - Ct ALK 3' | Final result |
| --- | --- | --- | --- | --- | --- | --- | --- | --- |
| #1 | 73 | F | adenocarcinoma | ND | 10,3 | -1,5 | 0,3 | *EML4-ALK (E6ins33;A17)^†^* |
| #2 | 52 | M | ND | ND | 10,1 | -0,9 | 2,8 | *WT* |
| #3 | 77 | F | ND | ND | 9,3 | 1 | -2,5 | *KRAS G12C* |
| #4 | 63 | M | adenocarcinoma | ND | 8,9 | 3 | -2,6 | *KRAS G12V* |
| #5 | 64 | F | ND | ND | 8 | 8,2 | -0,3 | *KIF5B-ALK (K17;A20)* |
| #6 | 56 | F | ND | ND | 7,9 | -0,5 | 0,7 | *EML4-ALK* *(E6ins33;A18), (E6ins33;A17),  (E6;A18), (E6;A17) ^†^* |
| #7 | 58 | M | adenocarcinoma | ND | 7,9 | 1,7 | -2,3 | *WT* |
| #8 | 63 | M | ND | ND | 7,8 | 7,2 | 0,1 | *ND* |
| #9 | 81 | F | adenocarcinoma | ND | 7,8 | 6,7 | 0,5 | *WT* |
| #10 | 62 | F | adenocarcinoma | ND | 7,5 | 7,7 | -0,5 | *EML4-ALK (E5del10;del44A20) ^†^* |
| #11 | 60 | M | adenocarcinoma | smoker | 7,4 | 1,6 | -4,1 | *KRAS G12D* |
| #12 | 61 | F | ND | ND | 6,8 | 5,6 | 1,1 | *EML4-ALK (E2;A20)* |
| #13 | 77 | M | adenocarcinoma | ND | 6,8 | 2,1 | -0,6 | *KRAS G12D* |
| #14 | 33 | F | adenocarcinoma | never smoker | 6,8 | 6,8 | 0,4 | *HIP1-ALK (H30;A20) ^†^* |
| #15 | 50 | F | adenocarcinoma | never smoker | 6,6 | 2,2 | -1,2 | *KRAS G12D* |
| #16 | 54 | M | adenocarcinoma | ND | 6,4 | 1,4 | -2,3 | *KRAS G12C* |
| #17 | 69 | F | adenocarcinoma | ND | 6,4 | 6,8 | 0,3 | *DCTN1-ALK (D26;A20)* |
| #18 | 69 | M | adenocarcinoma | ND | 6,3 | 1,7 | -3,4 | *NRAS Q61L* |
| #19 | 65 | F | ND | ND | 6 | 0,1 | -2,5 | *KRAS G12D* |
| #20 | 16 | M | ND | ND | 5,9 | 5,1 | -1,6 | *EML4-ALK (E2;A20)* |
| #21 | 52 | F | adenocarcinoma | never smoker | 5,9 | 1,8 | -3,1 | *NRAS Q61L* |
| #22 | 57 | M | ND | ND | 5,8 | 2,5 | -2,2 | *ND* |
| #23 | 58 | M | adenocarcinoma | smoker | 5,7 | 2,4 | -3 | *KRAS G12V* |
| #24 | 65 | F | adenocarcinoma | smoker | 5,5 | 2,5 | -2,2 | *WT* |
| #25 | 68 | M | adenocarcinoma | ND | 5,4 | 2 | -2,3 | *KRAS G12C* |
| #26 | 80 | F | adenocarcinoma | ND | 5,4 | 3,1 | -2,7 | *EGFR V769L* |
| #27 | 70 | F | adenocarcinoma | ND | 5,1 | 1,4 | -2,4 | *WT* |
| #28 | 57 | M | adenocarcinoma | ND | 5 | 2,3 | -2,3 | *KRAS G12D* |
| #29 | 62 | F | adenocarcinoma | ND | 4,9 | 3,2 | -1,2 | *WT* |
| #30 | 65 | F | adenocarcinoma | ND | 4,8 | 1,1 | -2,6 | *KRAS G12C* |
| #31 | 48 | M | adenocarcinoma | smoker | 4,7 | 2,6 | -2,4 | *KRAS G12C* |
| #32 | 66 | M | adenocarcinoma | never smoker | 4,1 | 1,7 | -2,2 | *KRAS G12W* |
| #33 | 38 | M | adenocarcinoma | ND | 4 | 0 | -2,2 | *KRAS G12C* |
| #34 | 69 | F | adenocarcinoma | ND | 4 | 4,2 | -0,7 | *KRAS G12V* |
| #35 | 48 | F | adenocarcinoma | never smoker | 3,5 | 4,5 | -0,3 | *WT* |
| #36 | 57 | F | adenocarcinoma | ND | 3,4 | 1 | -1,2 | *WT* |
| #37 | 66 | M | adenocarcinoma | ND | 3,4 | 2,4 | -2 | *WT* |
| #38 | 72 | F | adenocarcinoma | smoker | 3,4 | 2 | -1,7 | *CCDC6-RET (C1;R12)* |
| #39 | 54 | M | adenocarcinoma | ND | 3,4 | 3,9 | -2,3 | *WT* |
| #40 | 73 | M | ND | ND | 2,8 | 1,7 | -1,7 | *WT* |
| #41 | 78 | F | adenocarcinoma | ND | 2,2 | 1 | -0,9 | *KRAS G12V* |
| #42 | 47 | M | ND | ND | 1,5 | 2,5 | -2,4 | *WT* |
| #43 | 66 | F | adenocarcinoma | smoker | 1,4 | -0,2 | -0,7 | *WT* |
| #44 | 57 | F | adenocarcinoma | ND | 1,4 | 2,3 | -0,8 | *KRAS G12D* |
| #45 | 64 | M | adenocarcinoma | smoker | 0,6 | -0,1 | -0,3 | *WT* |
| #46 | 50 | M | adenocarcinoma | ND | 0,2 | 2,6 | -0,1 | *WT* |
| #47 | 73 | F | adenocarcinoma | ND | 0 | -0,2 | 0,4 | *WT* |
| #48 | 76 | F | adenocarcinoma | never smoker | -0,2 | 1,9 | -0,8 | *WT* |
| #49 | 65 | M | adenocarcinoma | ND | -0,6 | 0,8 | 1,4 | *KRAS G12C* |
| #50 | 60 | M | adenocarcinoma | ND | -0,6 | 0,8 | 1 | *KRAS G12C* |

^†^Detailed NGS results for these four samples are provided in the Table A.9. Ct – PCR cycle threshold; F – female; M – male; ND – no data available.

Table S9. Detailed NGS results for samples with *ALK* rearrangements

|  | Junction Read Count | Left Gene | Left Breakpoint | Right Gene | Right Breakpoint | Frameshift | Breakpoint’s details (cDNA) to **Ensembl Canonical (^EC^)** |
| --- | --- | --- | --- | --- | --- | --- | --- |
| #1 | 189 | EML4  ENSG00000143924.19 | chr2:42264951:+  **int 6-7 –** **ENST00000318522.10^EC^**  ex 7 – ENST00000401738.3 | ALK  ENSG00000171094.18 | chr2:29227074:-  **ex 18 – ENST00000389048.8^EC^** | inframe | *EML4-ALK (E6ins33^†^;A18)* |
|  | …taccaaaagttaccaaaactgcagacaaGCATAAAGATGTCATCATCAACCAAGcaaaaatgtcaactcgcgaaaaaaacagccaagTGATGGAAGGCCACGGGG…  EML4 exon 5, 6 (**ENST00000318522.10^EC^**, ENST00000401738.3), full sequence (33bp) of EML4 exon 7 from ENST00000401738.3 \| ALK ex18 **ENST00000389048.8^EC^** | | | | | |  |
| #6 | 320 | EML4  ENSG00000143924.19 | chr2:42264951:+  **int 6-7 –** **ENST00000318522.10^EC^**  ex 7 – ENST00000401738.3 | ALK  ENSG00000171094.18 | chr2:29227074:-  **ex 18 –** **ENST00000389048.8^EC^** | inframe | *EML4-ALK (E6ins33^†^;A18)* |
|  | …taccaaaagttaccaaaactgcagacaaGCATAAAGATGTCATCATCAACCAAGcaaaaatgtcaactcgcgaaaaaaacagccaagTGATGGAAGGCCACGGGG…  EML4 exon 5, 6 (**ENST00000318522.10^EC^**, ENST00000401738.3), full sequence (33bp) of EML4 exon 7 from ENST00000401738.3 \| ALK ex18 **ENST00000389048.8^EC^** | | | | | |  |
|  | 250 | EML4  ENSG00000143924.19 | chr2:42264951:+  **int 6-7 – ENST00000318522.10^EC^**  ex 7 – ENST00000401738.3 | ALK  ENSG00000171094.18 | chr2:29227672:-  **ex 17 – ENST00000389048.8^EC^** | inframe | *EML4-ALK*  *(E6ins33^†^;A17)* |
|  | …taccaaaagttaccaaaactgcagacaaGCATAAAGATGTCATCATCAACCAAGcaaaaatgtcaactcgcgaaaaaaacagccaagGCGGCAATGCAGCCTCAA…  EML4 exon 5, 6 (**ENST00000318522.10^EC^**, ENST00000401738.3), full sequence (33bp) of EML4 exon 7 from ENST00000401738.3 \| ALK ex17 **ENST00000389048.8^EC^** | | | | | |  |
|  | 196 | EML4  ENSG00000143924.19 | chr2:42264731:+  **ex 6 – ENST00000318522.10^EC^** | ALK  ENSG00000171094.18 | chr2:29227074:-  **ex 18 – ENST00000389048.8^EC^** | inframe | *EML4-ALK (E6;A18)* |
|  | …AATTAATACCAAAAGTTACCAAAACTGCAGACAAgcataaagatgtcatcatcaaccaagTGATGGAAGGCCACGGGGAAGTGAATATTAAGCATTATCTAAACTG…  EML4 exon 5, 6 **ENST00000318522.10^EC^** \| ALK exon 18 **ENST00000389048.8^EC^** | | | | | |  |
|  | 57 | EML4  ENSG00000143924.19 | chr2:42264731:+  **ex 6 – ENST00000318522.10^EC^** | ALK  ENSG00000171094.18 | chr2:29227672:-  **ex 17 – ENST00000389048.8^EC^** | inframe | *EML4-ALK (E6;A17)* |
|  | …AATTAATACCAAAAGTTACCAAAACTGCAGACAAgcataaagatgtcatcatcaaccaagGCGGCAATGCAGCCTCAAACAATGACCCCGAAATGGATGGGGAAGA…  EML4 exon 5, 6 **ENST00000318522.10^EC^** \| ALK exon 17 **ENST00000389048.8^EC^** | | | | | |  |
| #10 | 24 | EML4  ENSG00000143924.19 | chr2:42263296:+  **ex5 – ENST00000318522.10^EC^** | ALK  ENSG00000171094.18 | chr2:29223484:-  **ex 20 – ENST00000389048.8^EC^** | frameshift | *EML4-ALK (E5del10; del44A20)* |
|  | …aattgtcgaaaataccttcaacacccaaattaataccaaaagttaccaaaaCTGCAGAGCCCTGAGTACAAGCTGAGCAAGCTCCGCACCTCGACCATCATGACCG…  deletion 10 bp at the 3’-end of EML4 exon 5 **ENST00000318522.10^EC^** \| deletion 44 bp at the 5’-end ALK exon 17 **ENST00000389048.8^EC^** | | | | | |  |
| #14 | 157 | HIP1  ENSG00000127946.17 | chr7:75539323:-  **ex30 – ENST00000336926.11^EC^** | ALK  ENSG00000171094.18 | chr2:29223528:-  **ex 20 – ENST00000389048.8^EC^** | inframe | *HIP1-ALK (H30;A20)* |
|  | …ctgggagagcttcggaaaaagcactacgagcttgctggtgttgctgagggctgggaagaagTGTACCGCCGGAAGCACCAGGAGCTGCAAGCCATGCAGATGGAGC…  HIP1 exon 30 **ENST00000336926.11^EC^** \| ALK exon 20 **ENST00000389048.8^EC^** | | | | | |  |

^†^Full sequence of EML4 exon 7 ENST00000401738; EC - Ensembl Canonical transcript

**Supplemental Figures**

**
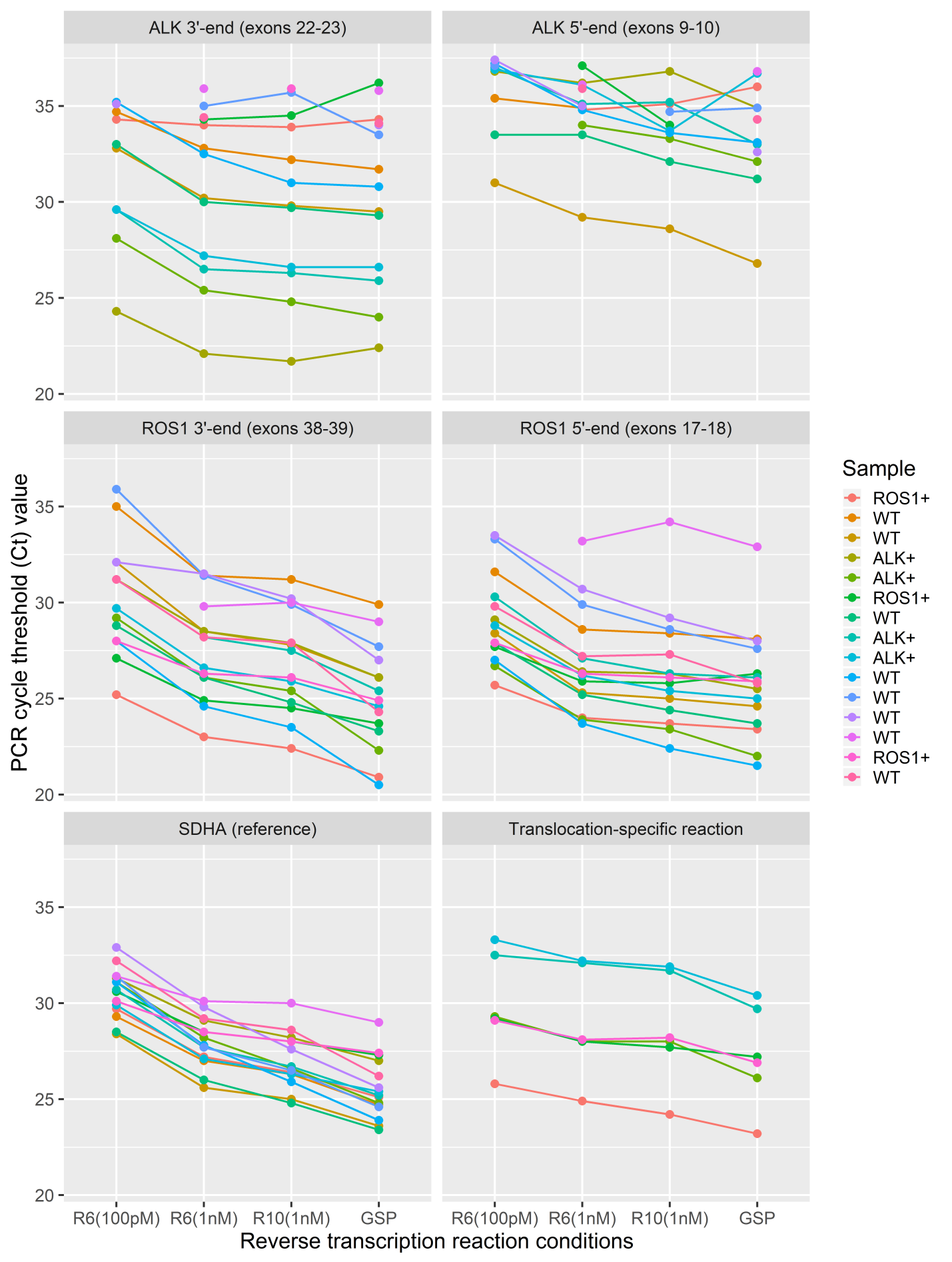
**

**Fig. S1.** Changes in PCR cycle threshold (Ct) values as a result of different reverse transcription priming strategies in individual samples. R6 – random hexamer primers; R10 – random decamers primers; GSP – gene-specific primers. Amount of primers per single reverse transcription reaction is given in parentheses.


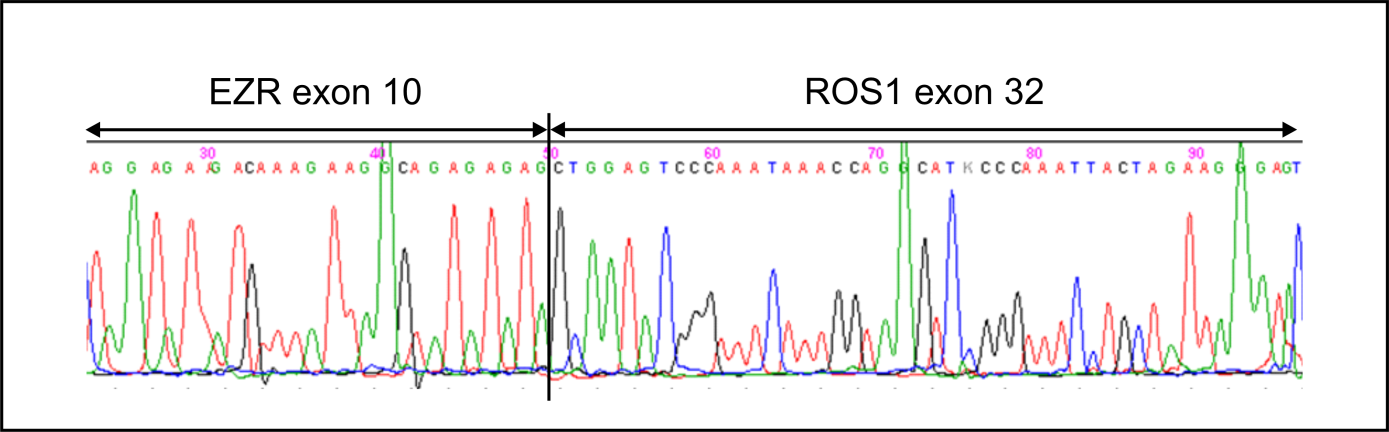


**Fig. S2.** Verification of a new translocation, *EZR-ROS1 (E10;R32)*, by Sanger sequencing.

**
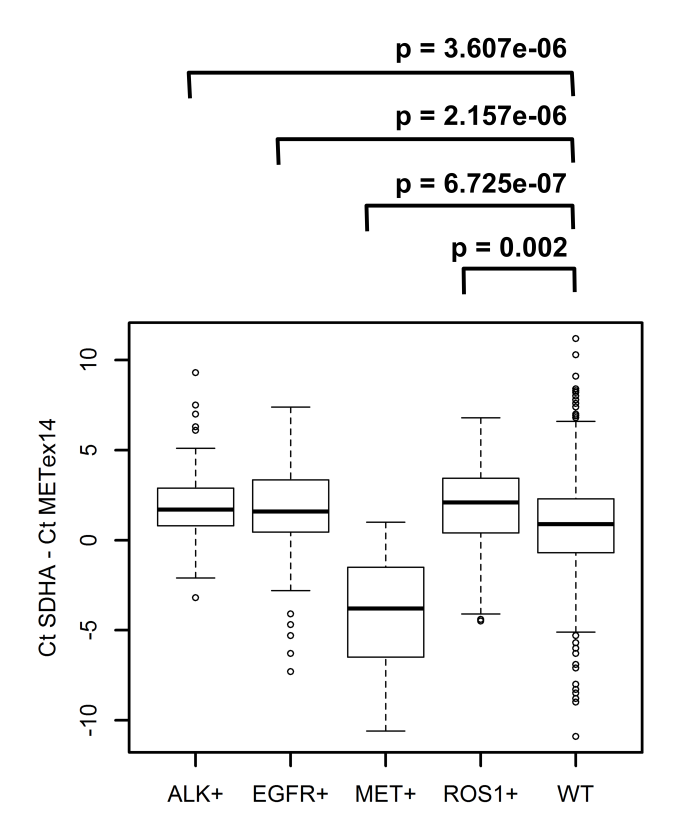
**

**Fig. S3.** *MET* exon 14 expression relative to the reference gene, *SDHA*, in samples positive for *ALK* fusions, *EGFR* mutations, *MET* exon 14 skipping, *ROS1* fusions and samples without these mutational events. Mann-Whitney U test p-values are provided at the top of the figure. Wild type allele *MET* expression is low in samples with *MET* exon 14 skipping, as it was shown earlier [Mitiushkina N.V. et al. Biochimie 2019, 165:267-274], while it is significantly higher in *ALK*-, *EGFR*- and *ROS1*-positive samples, compared to wild-type samples.
